# Graves’ disease: moving forwards

**DOI:** 10.1136/archdischild-2022-323905

**Published:** 2022-07-13

**Authors:** Laura C Lane, Claire Louise Wood, Tim Cheetham

**Affiliations:** 1 Translational and Clinical Research Institute, Newcastle University, Newcastle upon Tyne, UK; 2 Paediatric Endocrinology, Great North Children's Hospital, Newcastle upon Tyne, UK

**Keywords:** endocrinology, adolescent health, biochemistry, genetics

## Abstract

Graves’ disease is a rare disorder that continues to present clinicians and families with a series of challenges. There are no new established treatments for children or adolescents, but the outcomes of recent clinical trials and meta-analyses have helped clinicians to prepare families for the road ahead. We have a more refined understanding of how to administer antithyroid drugs, which one to use and how long to treat the young person. We also have a greater insight into how best to reduce any risks associated with surgery and radioiodine. We understand more about long-term outcomes and their determinants and have greater awareness about the impact of the disease and its treatment on quality of life. A holistic approach to management is key to supporting and counselling young people and their families about the diagnosis and management options. In this review, we will discuss the recent literature and reflect on how this should be translated into clinical practice.

## Introduction

Graves’ disease (GD) is an autoimmune disorder that develops because thyroid receptor autoantibodies (TRAbs) stimulate the thyroid-stimulating hormone (TSH) receptor (TSHR) on the thyroid gland. The interaction between antibody and receptor results in excessive thyroid hormone secretion (hyperthyroidism). GD is the most common cause of hyperthyroidism in children and adolescents, with a reported incidence of 0.9 per 100 000 in patients under 15 years of age in the UK population.^1^ The worldwide incidence of paediatric GD appears to be rising, with global incidences reported between 1.5 and 6.5 per 100 000.^2–4^ It can occur at any stage of childhood, although it tends to increase in frequency with age.

The conventional treatment options of antithyroid drugs (ATDs), radioiodine (RAI) or surgery have remained largely unchanged for many years, despite most young people relapsing after a course of ATD or requiring lifelong thyroid hormone replacement after definitive treatment (RAI or surgery). On the surface, the picture appears rather bleak and yet there has been recent progress in terms of our understanding of how best to manage young people with GD, and the demand for new therapeutic options is leading to the emergence of novel immunomodulatory approaches to treatment.

This review has focused primarily on articles published in the last 10 years including systematic reviews, which have provided additional insights into the management and outcome of paediatric GD. The management of neonatal thyrotoxicosis is beyond the scope of this review.

## Diagnosing GD

### Clinical

**Figure 1:**
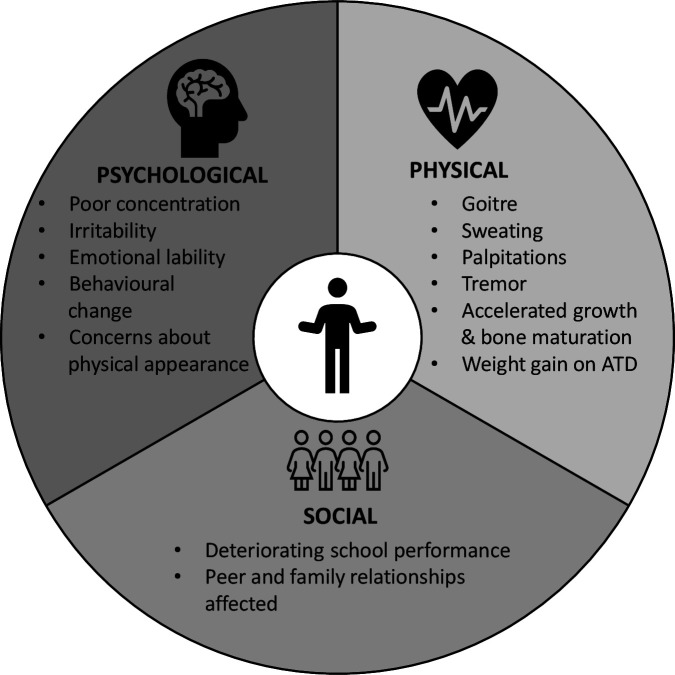
Key factors to consider when diagnosing and managing paediatric Graves’ disease. ATD, antithyroid drug.

The physical and psychological manifestations of GD reflect a state of hyperthyroidism and can include goitre, sweating, palpitations, tremor, irritability, behavioural changes, emotional lability, poor concentration, and accelerated growth and bone maturation.^5^ Extrathyroidal manifestations involving the eyes (Graves’ orbitopathy (GO)) and skin (pretibial myxoedema) can occur in children, although they are more commonly seen in adults. Importantly, there may also be social or academic concerns (figure 1). These symptoms can be insidious in nature, and due to the rarity of paediatric GD, are often mistakenly attributed to a primary psychological condition or disorders of the cardiovascular or gastrointestinal systems. Thus, there is the potential for a delay in diagnosis which can be detrimental in the context of a developing child or an adolescent who is undertaking examinations.

### Biochemical/immunological

The diagnosis of GD requires an assessment of both biochemical and immunological parameters. Thyroid function tests are characterised by a suppressed TSH with elevated free triiodothyronine (FT3)±free thyroxine (FT4) concentrations. An elevated FT3 level is a more sensitive marker of GD than an elevated FT4.^5^ The presence of an elevated TRAb titre is a key component when making a diagnosis of GD. There are two assays available to measure TRAb: the TSH-binding inhibition immunoglobulin assay which will gauge TSHR-specific antibodies, irrespective of whether they are stimulatory or not, and the thyroid-stimulating immunoglobulin bioassay that measures only the stimulatory antibodies. Both TRAb and anti-thyroperoxidase (TPO) antibodies should be measured because if TRAb levels are normal but TPO is elevated, then thyrotoxicosis may reflect the transient release of excess thyroid hormone from a thyroid gland where the main pathological process is destruction rather than stimulation. This is sometimes referred to as Hashitoxicosis. If both TRAb and TPO antibodies are absent, then non-autoimmune causes such as nodular thyroid disease or McCune-Albright syndrome are more likely.

### Imaging

In most cases, the biochemical and immunological findings will be sufficient to diagnose GD. Imaging should, however, be considered in cases where thyroid antibodies are negative or where there is a nodule or nodularity apparent on thyroid gland palpation. Ultrasonography is preferred over scintigraphy to avoid radiation exposure.^6^ Thyroid ultrasonography with Doppler blood flow assessment often displays an enlarged, hyperechoic, hypervascular thyroid gland, while scintigraphy shows uniform uptake of isotope and diffuse enlargement of both thyroid lobes. Imaging will also help identify the non-autoimmune causes of hyperthyroidism, such as an autonomous ‘hot’ nodule.

## Why do people develop GD?

The loss of immune self-tolerance to the TSHR is central to the pathogenesis of GD. The mechanisms that lead to this loss of tolerance result from a complex interplay of environmental factors and genetic/epigenetic susceptibility. Recently, the microbiome has also been implicated.^7^

### Environmental factors

The rising trend in the incidence of GD, alongside other autoimmune conditions, has been hypothesised to arise from underlying environmental factors such as infection and diet.^4^ The key environmental risk factors associated with GD include bacterial/viral infections, excess iodine intake, selenium and vitamin D deficiency, smoking and stress.^8^ Some of these factors, such as iodine excess, drive thyroid autoimmunity whereas others may induce it. Environmental exposures and infections are hypothesised to induce thyroid autoimmunity by ‘molecular mimicry’ (cross-reactivity between microbial antigens or exogenous agents and the TSHR) or ‘bystander activation’ (non-antigen-specific activation of autoreactive lymphocytes), turning a defensive immune response into autoimmunity.^9^ Environmental factors have also been proposed to trigger thyroid autoimmunity through epigenetic dysregulation modulating gene expression in susceptible individuals.^10^

### Genetic factors

Familial studies suggest that around 63% of GD predispositions are attributable to genetic factors.^11^ To date, several genetic loci have been associated with GD including immune-related genes (eg, *MHC, CTLA4, PTPN22*, *CD40*, *FCRL3, PD-1, BAFF*) and thyroid-specific genes (eg, *TSHR*, *TG*).^12 13^ There is growing evidence for the influence of non-coding genetic variants in autoimmune disease, including thyroid autoimmunity.^14^ Indeed, the human leucocyte antigen complex P5, which encodes a long non-coding RNA, is independently associated with GD susceptibility and younger age of onset.^15^ Other non-coding RNAs, such as microRNAs, are associated with GD and proposed as potential diagnostic and prognostic biomarkers.^16^

### Microbiome

Recent studies have hypothesised that intestinal dysbiosis (imbalance of gut microorganisms) caused by factors such as diet, smoking, stress, antibiotic use and toxins influence the production of cytokines and other metabolites, leading to the disruption of immune homeostasis and loss of tolerance in GD.^17^ A small study in adults with GD demonstrated a reduced risk of hyperthyroid relapse in individuals randomised to take a probiotic alongside their ATD.^18^ Further work on a larger scale is required to validate these findings and so pave the way for future microbiota-targeting therapeutics.

## Natural history of GD

Studies in adults have shown that the pathogenic TRAb antibodies can come and go with no apparent associated thyroid dysfunction or clinical phenotype.^19^ Spontaneous remission of GD is reported in adults but is probably even more uncommon in children. Children with established GD should be treated so that serious complications such as thyroid storm or life-threatening arrhythmias can be prevented. Hypothyroidism will develop in some patients with GD in the longer term, potentially due to the presence of destructive TPO antibodies from coexisting Hashimoto’s disease. Data in younger people are lacking but a quarter of adult patients who remit after ATD (see below) go on to require thyroxine replacement.^20^

## Management

Key considerations when managing a young person with GD are summarised in box 1. The general approach involves a course of ATD medication. If this is not possible or if this fails to result in remission, definitive treatment (RAI or thyroidectomy) needs to be considered. Ultimately, the main objectives when treating GD are restoration of euthyroidism in the short term and preservation of endogenous thyroid function in the long term without the need for medication. Unfortunately, with the currently available therapies, this is not a realistic prospect for many children.

Box 1 **Key points when managing paediatric Graves’ disease**

- Use CBZ or MMI, avoid PTU.
- CBZ is commenced in a dose of 0.25–0.75 mg/kg/day (0.15–0.5 mg/kg/day MMI).
- A dose at the lower end of this range will be appropriate if thyroid hormone excess is at the less profound end of the spectrum.
- DT is the preferred method of ATD treatment, although BR may be helpful if there is persistent relapse on small-dose ATD.
- Beta-blockade is recommended in patients presenting with signs of marked thyroid hormone excess.
- An FBC and LFTs should be checked at baseline.
- Be guided by thyroid hormone concentrations (not TSH) in first few months to alter dosage.
- Counsel families about the risk of excessive weight gain when ATD is commenced.
- A TRAb level should be checked before antithyroid medication is stopped—remission is much more likely in the context of a normal value.
- ATD is normally administered for at least 3 years. Longer courses (≥5 years) should be considered if the likelihood of remission is low.
- Patients who relapse after a course of ATD can return to ATD or choose definitive treatment (RAI/surgery).
- If there are academic concerns, then consider liaising with the child’s school to ensure specific support is provided as required.

ATD, antithyroid drug; BR, block and replace; CBZ, carbimazole; DT, dose titration; FBC, full blood count; LFTs, liver function tests; MMI, methimazole; PTU, propylthiouracil; RAI, radioiodine; TRAb, thyroid receptor autoantibodies; TSH, thyroid-stimulating hormone.

### Initial management

Not all thyrotoxicosis or indeed autoimmune thyroid disease with associated thyrotoxicosis reflects GD. It is therefore important to establish the underlying mechanism at an early stage. If TRAb concentrations are elevated, then the thyrotoxicosis is due to GD and is unlikely to remit in the near future. In the absence of TRAb, the presence of TPO antibodies can suggest underlying thyroiditis which can be self-limiting. Hence, it may be appropriate to monitor thyroid function tests for a few weeks before intervening with ATD treatment if the young person is not significantly unwell in the context of a low TRAb titre.^6^ If the child has GD then ATD should be commenced to avoid the negative physical and psychological effects of prolonged thyroid hormone excess.

Children with signs of GO should be reviewed by an orbital specialist, preferably in a combined (ophthalmologist/clinician) thyroid eye clinic.^6^

### Beta blockade

A beta-blocker (eg, propranolol or atenolol) should be prescribed for patients presenting with signs of moderate to severe thyroid hormone excess. This can be stopped once the patient is biochemically euthyroid.^6^ Beta-blockers are contraindicated in patients with asthma.

### Thyroid storm

A thyroid storm (acute thyrotoxic crisis) may arise in someone with undiagnosed or poorly controlled GD and can occur very occasionally following RAI therapy. A beta-blocker is indicated in the rare case of a thyroid storm, where it is typically used alongside ATD, a glucocorticoid and iodine.

### Antithyroid medication

The ATD used to treat GD in children is the thionamide carbimazole (CBZ) or its active metabolite methimazole. CBZ does not need to be given more than once daily. Propylthiouracil (PTU) was previously used but should now be avoided in children because of the risk of liver failure.^21^ A framework for managing patients with ATD, including recommended starting doses,^6^ is presented in box 1.

Thionamides block thyroid hormone synthesis by inhibiting the TPO-mediated iodination of tyrosine residues in thyroglobulin, a vital step in the synthesis of thyroxine.^22^ In addition, both direct and indirect immunomodulatory effects have been proposed, but it is challenging to distinguish between the direct effect of ATD on the immune system versus the indirect beneficial effect of rendering the patient euthyroid. Furthermore, ATD has been shown to possess antioxidant activity by reducing the oxidative stress caused by reactive oxygen species.^22^

Both hyperthyroidism and ATD can be associated with a low white cell count and liver dysfunction. Before commencing ATD, a full blood count (FBC) and liver function should therefore be checked so that the mechanism behind any subsequent changes can be placed into context.^6^

#### ATD side effects

The potential side effects of ATD treatment and their frequencies are outlined in table 1. Approximately 20% of children on ATD will develop at least one side effect/adverse event.^23^ Younger children appear to be at greater risk, with adverse reactions reported in up to 71% of prepubertal children.^24^ The most frequent side effects involve cutaneous skin reactions that either resolve spontaneously or with a course of antihistamines.^25^ Rarely, hepatic dysfunction can develop which is cholestatic in nature, and unlike with PTU, will resolve on stopping treatment.^26^ Most side effects occur within the first 3 months of treatment,^27^ although the rare but potentially fatal side effect agranulocytosis (absolute neutrophil count of <0.5×10^9^/L) can arise after many years.^28^

**Table 1:** Frequency of antithyroid drug adverse events

Frequency	Adverse event
Common (1.1%–11%)	Skin rash Arthralgia/myalgia Neutropenia/leucopenia
Rare (0.2%–1%)	Liver transaminase elevation Headache Gastrointestinal complaints Agranulocytosis Hair loss Fever Sore throat
Very rare (<0.1%)	Aplastic anaemia Thrombocytopenia Cholestatic jaundice/hepatitis

It is essential that when ATD is prescribed, the young person and their family are counselled about the importance of stopping the medication in the event of a sore throat or fever until they have an FBC checked. If agranulocytosis develops, alternative treatment should be initiated. A relative neutropenia (0.5–1.5×10^9^/L) can be monitored closely with once or biweekly measurements.^6^

#### Dose titration or block and replace?

There are two main strategies when administering ATD therapy. A ‘dose titration’ (DT) method can be used where the ATD dose is titrated against circulating thyroid hormone concentrations. Alternatively, the ‘block and replace’ (BR) approach can be used which involves completely blocking thyroid hormone production while adding exogenous thyroxine supplementation. Both are used in the UK although data from a randomised UK trial showed no difference between the two approaches in terms of biochemical stability.^29^ There is some evidence to suggest that higher doses of ATD, as used with BR, may increase the likelihood of drug side effects and recent European Thyroid Association (ETA) guidelines suggest using the DT approach in most instances.^6^ It is also possible that the simpler DT regimen (one medication rather than two) facilitates earlier resolution of the hyperthyroid state.^29^ There is still an argument for using the BR approach in patients who, for example, repeatedly undergo a biochemical relapse while on a reducing dose of ATD.

#### Monitoring response to ATD treatment

In the initial period of treatment, thyroid hormone concentrations should be checked at least every 4 weeks until thyroid hormone concentrations have normalised, after which monitoring can potentially be reduced to every 2–3 months.^6^ TSH levels can remain low for a significant period after ATD has been commenced, with approximately one-third of patients still having a suppressed TSH at 6 months after diagnosis.^29^ This highlights the importance of using thyroid hormone concentrations (FT3/FT4) to make decisions about altering the dose of ATD in the initial months. After a month of treatment, around 50% of patients will have normalised their FT4 concentrations, rising to 90% by 6 months. Younger, prepubertal children often present with more severe thyrotoxicosis and can take longer to normalise thyroid hormone levels than older children.^24^ Prepubertal children can also present with an isolated elevated T3 (T3 toxicosis), highlighting the importance of measuring FT3.^24^ Excessive weight gain can occur as euthyroidism is restored.^29^ Families should be made aware of this possibility and pertinent advice provided.

#### Duration of treatment

The overall remission rate after 2 years of ATD in young people is only 20%–30%.^30^ There are several clinical and biochemical factors associated with an increased risk of relapse (table 2). These factors can be discussed when counselling the family about the likelihood of remission following ATD. The duration of ATD is one of the few modifiable risk factors, with a recent systematic review reporting some evidence of longer treatment duration increasing remission rate (table 3).^30^ It is important to note that studies documenting higher remission rates with longer-term ATD treatment are relatively small and occur in the context of a variety of patient populations with different underlying risk factors.

**Table 2:** Factors associated with an increased risk of relapse in Graves’ disease

Parameter type	Factor
Clinical	Younger age Male* Non-Caucasian ethnicity Large goitre No other autoimmune conditions
Biochemical	More severe thyrotoxicosis at diagnosis
Immunological	High TRAb level at diagnosis High TRAb level on stopping ATD
Treatment course	Shorter duration of ATD treatment
Genetic polymorphisms*	eg, *HLA* subtypes (DB1*02, DQA1*05 and DRB1*03) and *PTPN22 (rs2476601*)

*In adult studies only.

ATD, antithyroid drug; HLA, human leucocyte antigen; PTPN22, protein tyrosine phosphatase non-receptor type 22; TRAb, thyroid receptor autoantibodies.

**Table 3:** Pooled remission rates from studies in paediatric patients with Graves’ disease based on antithyroid treatment duration (adapted from the systematic review by Lieshout *et al*)^30^

Treatment duration, years	Patients, n	Remission rate, % (pooled)
1.5–2	620	23.7
2.5–5	1826	31
5–6	174	43.7
9	28	75

The ETA recommends treatment for at least 3 years so that the likelihood of remission can be optimised, although the relationship between duration and outcome is not particularly robust. When considering whether ATD treatment should be stopped, it is essential that a TRAb level is measured because if TRAbs are elevated, the chances of remission are low.^31^ Patients who relapse tend to do so within the first 12 months after stopping treatment and so withdrawal of ATD should be avoided during periods where a relapse would be particularly detrimental, such as prior to academic examinations. One signal that might indicate a trial off ATD therapy is warranted would be a normal TRAb titre in a patient who has developed a raised TSH on a low ATD dose.

### Longer-term ATD use

Patients can be maintained in the long term on low-dose ATD, but there is still the very small risk of agranulocytosis and, as alluded to above, relapse can still occur on stopping.

## Definitive treatment

Many young people will ultimately experience a relapse of their GD. In this scenario, or in cases where ATD is unsuitable or not tolerated, then definitive treatment is warranted. The aim of RAI or a total thyroidectomy is to ablate or remove all functional thyroid tissue and so will necessitate lifelong levothyroxine replacement.

### RAI therapy

RAI is administered orally in the form of a capsule or solution. It is rapidly incorporated into the thyroid gland with radioactive emissions destroying the thyroid cells over a period of a few weeks and resulting in hypothyroidism.

European guidelines advise that ablative doses of RAI should be used in children to achieve relatively rapid hypothyroidism and minimise the future risk of relapse.^6^ RAI should be avoided in the very young (<5 years) because of the greater theoretical risk of later malignancy. However, observational studies including a 36-year follow-up study of children aged between 3 and 19 years found RAI to be a safe and effective therapy.^32 33^ Studies examining the risk of malignancy after RAI are complicated by the fact that hyperthyroidism itself may increase the risk of solid malignancies.^34^ A recent review concluded that RAI is similar from a safety perspective when compared with treatment with ATD or surgery.^34^

Adult studies have shown that RAI can trigger the development or exacerbation of pre-existing GO and so this modality should be avoided in the relatively rare paediatric patient with active GO and patients without active GO should be monitored carefully afterwards.^35^ Occasionally, a repeat dose of RAI may be required if complete thyroid ablation has not occurred, particularly in those with a large goitre for whom a thyroidectomy may be deemed more appropriate.

### Thyroidectomy

A thyroidectomy may be preferred over RAI in younger children, in the presence of a large goitre or if RAI is contraindicated. Mortality rates in young people undergoing thyroidectomy are very low (<0.1%), but a minority of patients will experience complications such as hypocalcaemia (22%) and recurrent laryngeal nerve injury (5.4%).^36^ These morbidities are usually transient but can be permanent.^36^ Prior to surgery, patients should be vitamin D replete to reduce the risk of postoperative transient hypocalcaemia.

Thyroidectomies performed by high-volume surgeons have better outcomes with less postoperative morbidity and so paediatric thyroidectomies should be undertaken at high-volume centres.^6^

## Quality of life

The diagnosis of GD can be daunting for the young person and their family, who potentially face years on medication without a high expectation of cure. The short-term and long-term impact of GD on the young person from a physical, psychological and social perspective should not be underestimated. Indeed, patients with GD who were treated in childhood or adolescence report a lower quality of life than their healthy peers, particularly in the psychosocial domain.^37^ In addition, adult studies have reported that thyroid hormone replacement is associated with a reduced quality of life compared with controls.^38^ This highlights the importance of considering additional support for young people with GD both from an academic and psychological perspective.

## Future directions

### Immunomodulation

Several immunotherapies are under investigation in adult GD, aiming to achieve euthyroidism without the need for ongoing therapy.^39^ A recent Medical Research Council-funded multicentre phase 2 trial investigated the use of an anti-CD20 monoclonal antibody, rituximab, alongside a 12-month course of ATD in 27 paediatric patients with GD.^40^ Almost half of the patients were in remission 1 year after stopping ATD (compared with 20%–30% usually seen 1–3 years after ATD cessation).

Whether immunomodulatory therapies alter the long-term outcome of GD as opposed to simply delaying relapse is unclear. The future management of paediatric GD will likely include a form of precision medicine, with treatments chosen based on individual clinical, immunological and genetic risk factors predicting likelihood of relapse.

## Conclusions

GD is a challenging condition for young people and their families, and the clinician has an important role in counselling them about the disease course, available treatment options and the likely long road ahead. It is important that a holistic approach is adopted that considers the needs of the young person as an individual, with extra support provided as needed. The emergence of potential novel therapeutic options is an exciting prospect for the future management of GD.
